# Prevalence of Distress Associated With Difficulty Controlling Sexual Urges, Feelings, and Behaviors in the United States

**DOI:** 10.1001/jamanetworkopen.2018.4468

**Published:** 2018-11-09

**Authors:** Janna A. Dickenson, Neil Gleason, Eli Coleman, Michael H. Miner

**Affiliations:** 1 Program in Human Sexuality, Department of Family Medicine and Community Health, University of Minnesota, Minneapolis

## Abstract

**Importance:**

The veracity, nomenclature, and conceptualizations of sex addiction, out-of-control sexual behavior, hypersexual behavior, and impulsive or compulsive sexual behavior are widely debated. Despite such variation in conceptualization, all models concur on the prominent feature: failing to control one’s sexual feelings and behaviors in a way that causes substantial distress and/or impairment in functioning. However, the prevalence of the issue in the United States is unknown.

**Objective:**

To assess the prevalence of distress and impairment associated with difficulty controlling sexual feelings, urges, and behaviors among a nationally representative sample in the United States.

**Design, Setting, and Participants:**

This survey study used National Survey of Sexual Health and Behavior data to assess the prevalence of distress and impairment associated with difficulty controlling sexual feelings, urges, and behaviors and determined how prevalence varied across sociodemographic variables. Participants between the ages of 18 and 50 years were randomly sampled from all 50 US states in November 2016.

**Main Outcomes and Measures:**

Distress and impairment associated with difficulty controlling sexual feelings, urges, and behavior were measured using the Compulsive Sexual Behavior Inventory–13. A score of 35 or higher on a scale of 0 to 65 indicated clinically relevant levels of distress and/or impairment.

**Results:**

Of 2325 adults (1174 [50.5%] female; mean [SD] age, 34.0 [9.3] years), 201 [8.6%] met the clinical screen cut point of a score of 35 or higher on the Compulsive Sexual Behavior Inventory. Gender differences were smaller than previously theorized, with 10.3% of men and 7.0% of women endorsing clinically relevant levels of distress and/or impairment associated with difficulty controlling sexual feelings, urges, and behavior.

**Conclusions and Relevance:**

The high prevalence of this prominent feature associated with compulsive sexual behavior disorder has important implications for health care professionals and society. Health care professionals should be alert to the high number of people who are distressed about their sexual behavior, carefully assess the nature of the problem within its sociocultural context, and find appropriate treatments for both men and women.

## Key Points

**Question:**

What is the prevalence among US men and women of the primary feature of compulsive sexual behavior disorder, distress and impairment associated with having difficulty controlling one’s sexual feelings, urges, and behaviors?

**Findings:**

In this survey study, we found that 8.6% of the nationally representative sample (7.0% of women and 10.3% of men) endorsed clinically relevant levels of distress and/or impairment associated with difficulty controlling sexual feelings, urges, and behaviors.

**Meaning:**

The high prevalence of such symptoms has major public health relevance as a sociocultural problem and indicates a significant clinical problem that should be recognized by health care professionals.

## Teaser

This survey study investigates the prevalence of distress and impairment associated with difficulty controlling one’s sexual feelings, urges, and behaviors among US men and women.

## Introduction

From Tiger Woods to Harvey Weinstein, news articles have conjectured that “sex addiction” is a growing and heretofore unrecognized “epidemic,”^1^ while the scientific community debates whether such a problem even exists. Although psychiatry has a long history of attempting to characterize hypersexuality, researchers and clinicians have disparate views regarding whether it represents a true psychiatric disorder or is merely indicative of a larger sociocultural problem (labeled as *out-of-control sexual behavior*^2^). Moreover, there has been considerable disagreement regarding conceptualization, etiology, and nomenclature (eg, *compulsive sexual behavior* [CSB],^3^ *hypersexual disorder,*^4^ *sexual addiction,*^5^ and *out-of-control sexual behavior*^2^).^6^ Symptom presentation also varies across conceptualizations, rendering the precise estimation of national prevalence difficult.^7^ Consequently, scientists’ ability to empirically examine the veracity of pop culture’s supposition that CSB is a “growing epidemic”^1^ remains limited.

Despite such lack of consensus regarding conceptualization and operationalization, all conceptualizations share a common feature: having substantial difficulty controlling one’s sexual feelings, urges, and behaviors that causes clinically significant levels of distress and/or impairment. This key feature forms the basis of the new classification of compulsive sexual behavior disorder (CSBD), which, for the first time, has gained recognition as a formal disorder in the *International Classification of Diseases, Eleventh Revision,* under the class of impulse control disorders.^7^ Specifically, CSBD is characterized by a persistent pattern of failure to control intense, repetitive sexual urges, which results in repetitive sexual behavior that causes marked distress or social impairment. Such distress and impairment includes neglecting social activities or personal health, repeatedly attempting to control sexual behavior unsuccessfully, and continuing to engage in sexual behavior despite adverse consequences or even when the individual derives minimal pleasure from his or her sexual activities.

Given the recency of the classification of CSBD and preceding absence of consistent definitions, we know of no systematic epidemiological studies of this disorder that have been conducted in the United States. Rough estimates of the perception of one’s sexual behavior being out of control have been obtained in other countries,^8^ and national prevalence in the United States has been estimated based on small samples.^4,7^ Such studies have indicated that relatively few individuals perceive their sexual behavior as out of control and experience distress and/or impairment due to their sexual behavior. In the United States, prevalence has been estimated to range from 1% to 6% in adults, with an expected male to female ratio from 2:1 to 5:1.^4,7^ Given the dearth of systematic epidemiological studies in the United States and debate surrounding definitions and specific symptom presentation, assessing the prevalence of distress and impairment associated with difficulty controlling one’s sexual feelings, urges, and behaviors provides the closest population-based estimate of CSBD available at this time.

The current study assesses the prevalence of this key feature in the United States by administering the Compulsive Sexual Behavior Inventory–13 (CSBI-13) to a nationally representative sample (Figure). The CSBI-13 was designed as a screening instrument to assess the severity of impulsive and compulsive sexual behavior.^9,10^ The current 13 items parallel the proposed criteria of CSBD and assess the severity of perceived difficulty controlling one’s sexual feelings, urges, and behavior and the degree of distress (feeling ashamed of sexual behavior, engaging in sexual behavior as a means of emotion regulation) and psychosocial impairment (social, interpersonal, and occupational consequences) associated with such behavior.^11^ Currently, the CSBI-13 is the only existing screening instrument with an established clinical cut point to accurately identify those who meet and do not meet criteria for the probable CSB syndrome 72% and 79% of the time, respectively.^11^ Based on prior US prevalence estimates of CSBD, we hypothesized that 1% to 6% of the population would meet the clinical cut point of the CSBI-13 and 20% to 30% of those who met the clinical cut point would be women.

**Figure.**
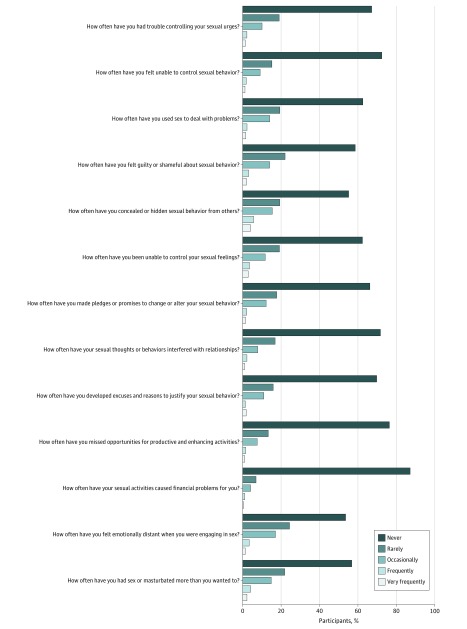
Frequency of Responses to Each Item on the Compulsive Sexual Behavior Inventory–13

## Methods

Data were collected as part of the population-based National Survey of Sexual Health and Behavior (NSSHB) following the American Association for Public Opinion Research (AAPOR) reporting guideline for survey studies. The NSSHB study was designed to examine sexual experiences among the US population between the ages of 18 and 50 years (mean [SD] participant age, 34.0 [9.3] years) and included individuals from all 50 states and the District of Columbia. Participants were recruited using KnowledgePanel (GfK Research) over a span of 2 weeks in November 2016 from the general population of adults who completed 1 of the previous waves of the NSSHB studies and from a fresh sample of the general adult population in the United States. Participants from both target groups were randomly recruited through probability-based sampling, and households were provided with access to the internet and hardware if needed.^12^ This method used the largest national sampling frame from which fully representative samples can be generated to produce statistically valid inferences for study populations. Of those who were sampled for the study, 51% (2594) pursued interest in the study by visiting the website where they could learn about the study. Of these individuals, 94% (2432) provided informed consent, and 95.6% (2324) of those who provided informed consent completed the CSBI-13. The NSSHB was approved by the Indiana University institutional review board.

### Measures

#### Compulsive Sexual Behavior Inventory

The CSBI-13 is a screening tool that assesses the core feature of CSBD: functional impairment and/or distress associated with difficulty controlling one’s sexual feelings, urges, and behaviors.^10^ The CSBI-13 has been shown to have adequate reliability, reliable criterion validity, and discriminant and convergent validity.^11^ Previous versions of the CSBI have been tested in various populations of adult men and women in the United States^13,14,15,16,17^ and in other countries.^17,18^ Participants rate each of the 13 items (Figure) on a 5-point scale ranging from 1 (never) to 5 (very frequently). The total scale score is computed by summing across items. A score of 35 or greater has been shown to be a sensitive and specific cut point for distinguishing individuals who meet criteria for the probable CSB clinical syndrome, which mirrors the proposed diagnostic criteria of CSBD.^11^ Because the CSBI-13 is a self-report screening tool that was created prior to the new classification of CSBD, a score of 35 or higher indicates a high probability of meeting diagnostic criteria and warrants further evaluation to ascertain the diagnosis of CSBD.

#### Sociodemographic Questions

Age, race/ethnicity, education, and household income were collected during GfK’s panel recruitment process. Income was reported categorically ranging from less than $5000 to $250 000 or higher. Given the number of ordinal categories, income was collapsed into the following categories: less than $25 000, $25 000 to $49 999, $50 000 to $74 999, $75 000 to $99 999, $100 000 to $150 000, and more than $150 000. Similarly, education level was collected categorically and was subsequently collapsed into the following categories: less than high school education, high school diploma or equivalent, some college or associate’s degree, bachelor’s degree, and master’s degree or higher. Respondents selected their ethnicity/race from the following options: white, non-Hispanic; black, non-Hispanic; multiple races, non-Hispanic; and Hispanic. During the survey, participants noted their gender as man, woman, transman, or transwoman. Because only 4 individuals identified as transgender, transgender individuals were categorized according to their gender identity. Participants also labeled their sexual orientation as heterosexual, bisexual, gay or lesbian, asexual, or something else. Those who identified as asexual or something else were combined, given the low frequency of these labels.

### Statistical Analysis

The prevalence of individuals who endorsed clinically relevant levels of distress and impairment associated with having difficulty controlling sexual feelings, urges, and behaviors was assessed by determining the proportion with 95% confidence intervals of individuals who scored 35 or higher on the CSBI-13 using descriptive statistics in SPSS statistical software version 22.0 (IBM). Characteristics among individuals who met and did not meet the clinical cut point of the CSBI-13 were presented as percentages (categorical variables) or means (continuous variables). To investigate differences in the proportion of individuals who met the clinical cut point of the CSBI-13 across various sociodemographic characteristics (eg, gender, race/ethnicity, and sexual orientation), χ^2^ statistics were calculated. Significant findings (2-sided *P* < .05) were further examined using binary regression with a log-link function to estimate differences in rate ratios across the various sociodemographic variables.

To correct sources of sampling and nonsampling error, the study sample was corrected with poststratification adjustments using demographic distributions from the most recent Current Population Survey from the US Census Bureau.^19^ These adjustments resulted in a panel base weight that was used in a probability proportional to size selection method for establishing the sample for the current study.^12^ All data presented in this study use these weights.

## Results

Participants (N = 2325) were between the ages of 18 and 50 years (mean [SD] age, 34 [9.26] years), with nearly equal numbers of male- and female-identified individuals (1174 [50.5%] female) (Table). Descriptive data on education indicated that 10.8% (251 participants) did not complete high school, 26.8% (622) completed high school, 30.7% (713) completed some college, 19.4% (450) obtained a bachelor’s degree, and 12.4% (289) obtained a professional degree. With regard to income, 19.7% (458) earned less than $25 000 and 41.0% (953) earned income more than $75 000. Concerning race and ethnicity, 19.8% (455) identified as Hispanic; 58.4% (1358) as white, non-Hispanic; 12.7% (296) as black, non-Hispanic; 1.6% (36) as multiple races, non-Hispanic; and 7.7% (179) as other, non-Hispanic. A total of 91.6% of participants (2128) described themselves as heterosexual, 4.4% (101) as bisexual, 2.6% (60) as gay or lesbian, and 1.4% (33) as something else. The Table delineates the distribution of sociodemographic characteristics across individuals who exhibited and did not exhibit clinically relevant levels of distress associated with their sexual urges and behavior, as well as differences in prevalence rates across various demographic variables.

**Table.** Demographic Characteristics of Survey Participants

Variable	No. (%)			χ^2^	*P* Value
	Total	CSBI-13 Score <35	CSBI-13 Score ≥35		
Gender					
Male identified	1150 (49.5)	1031 (48.6)	119 (59.2)	8.32	.004
Female identified	1174 (50.5)	1092 (51.4)	82 (40.8)		
Sexual orientation					
Heterosexual	2128 (91.6)	1968 (92.8)	161 (79.7)	43.14	<.001
Gay or lesbian	60 (2.6)	48 (2.3)	12 (5.9)		
Bisexual	101 (4.4)	81 (3.8)	20 (9.9)		
Other	33 (1.4)	24 (1.1)	9 (4.5)		
Race/ethnicity					
White, non-Hispanic	1358 (58.4)	1273 (60.0)	86 (42.8)	29.17	<.001
Black, non-Hispanic	296 (12.7)	253 (11.9)	43 (21.4)		
Other, non-Hispanic	179 (7.7)	157 (7.4)	21 (10.4)		
Multiple races, non-Hispanic	36 (1.6)	35 (1.6)	1 (0.5)		
Hispanic	455 (19.8)	405 (19.1)	50 (24.9)		
Education					
Less than high school	251 (10.8)	215 (10.1)	36 (17.8)	15.22	.004
High school or GED	622 (26.8)	577 (27.2)	45 (22.3)		
Some college	713 (30.7)	644 (30.3)	69 (34.2)		
Bachelor’s degree	450 (19.4)	419 (19.7)	31 (15.3)		
Professional degree	289 (12.4)	268 (18.0)	21 (10.4)		
Income, $					
<10 000	167 (7.2)	131 (6.2)	35 (17.5)	59.24	<.001
10 000-24 999	134 (5.8)	117 (5.5)	17 (8.5)		
25 000-49 999	443 (19.0)	416 (19.6)	26 (13.0)		
50 000-74 999	422 (18.1)	400 (18.8)	21 (10.5)		
75 000-99 999	357 (15.4)	320 (15.1)	38 (19.0)		
100 000-150 000	427 (18.4)	406 (19.1)	21 (10.5)		
>150 000	376 (16.2)	334 (15.7)	42 (21.0)		
Age, mean (SD), y	34.0 (9.3)	34.2 (9.29)	32.8 (8.94)	*t* = 1.94	.05

Abbreviations: CSBI-13, Compulsive Sexual Behavior Inventory–13; GED, General Educational Development.

### Prevalence Estimates

The prevalence rate of endorsing clinically relevant levels of distress and/or impairment associated with difficulty controlling sexual feelings, urges, and behaviors (CSBI-13 score ≥35) was 8.6% (95% CI, 7.5%-9.8%) (201 participants). Among men, 10.3% (119) endorsed clinically relevant levels of distress and/or impairment associated with difficulty controlling sexual feelings, urges, and behaviors, in comparison with 7.0% of women (82 participants). Although men were 1.54 (95% CI, 1.15-2.06) times more likely to endorse significant levels of distress associated with difficulty controlling sexual feelings, urges, and behaviors (χ^2^ = 8.32, *P* = .004), women accounted for nearly half (40.8%) of individuals who met the clinical screen cut point.

### Sociodemographic Differences

Significant differences in the likelihood of endorsing distress associated with difficulty controlling sexual feelings, urges, and behaviors across sociodemographic characteristics were further examined with logistic regression. With regard to income, we found that individuals with income less than $25 000 had higher odds of endorsing distress and impairment associated with difficulty controlling sexual feelings, urges, and behaviors compared with those with income of $25 000 to $49 999 (odds ratio [OR], 3.38; 95% CI, 2.06-5.55), $50 000 to $74 999 (OR, 4.01; 95% CI, 2.37-6.81), $75 000 to $99 999 (OR, 1.80; 95% CI, 1.15-2.82), $100 000 to $150 000 (OR, 4.08; 95% CI, 2.41-6.93), and more than $150 000 (OR, 1.67; 95% CI, 1.08-2.59). Additionally, those with incomes between $75 000 and $100 000 had higher odds of endorsing distress and impairment associated with difficulty controlling sexual feelings, urges, and behaviors compared with those with income between $25 000 and $50 000 (OR, 1.88; 95% CI, 1.12-3.16), $50 000 to $75 000 (OR, 2.23; 95% CI, 1.29-3.88), and $100 000 to $150 000 (OR, 2.27; 95% CI, 1.31-3.95). Similarly, those with income higher than $150 000 had higher odds compared with those with income between $25 000 and $50 000 (OR, 2.02; 95% CI, 1.22-3.36), $50 000 to $75 000 (OR, 2.40; 95% CI, 1.40-4.13), and $100 000 to $150 000 (OR, 2.44; 95% CI, 1.42-4.20). Regarding education, those with high school education (OR, 0.48; 95% CI, 0.30-0.76), some college (OR, 0.65; 95% CI, 0.42-0.99), bachelor’s degree (OR, 0.45; 95% CI, 0.27-0.74), or professional degree (OR, 0.47; 95% CI, 0.26-0.83) had lower odds of endorsing clinically relevant levels of distress and impairment associated with difficulty controlling sexual feelings, urges, and behaviors than individuals with less than high school education.

With respect to race/ethnicity, individuals who identified as black, other, and Hispanic were 2.50 (95% CI, 1.69-3.70), 2.02 (95% CI, 1.22-3.33), and 1.84 (95% CI, 1.27-2.65) times more likely, respectively, than white individuals to endorse clinically relevant levels of distress and impairment associated with difficulty controlling sexual feelings, urges, and behaviors. Finally, heterosexual individuals had lower odds of endorsing clinically relevant levels of distress and impairment associated with difficulty controlling sexual feelings, urges, and behaviors than those who identified as gay or lesbian, bisexual, or other. Relative to heterosexual individuals, gay or lesbian individuals were 2.92 (95% CI, 1.51-5.66) times more likely, bisexual individuals were 3.02 (95% CI, 1.80-5.04) times more likely, and individuals who identified as other were 4.33 (95% CI, 1.95-9.61) times more likely to endorse distress associated with difficulty controlling sexual feelings, urges, and behaviors. No other significant differences were found (*P* > .05 for all).

## Discussion

Has pop culture correctly assumed that CSB is an epidemic? Results suggest that a substantial proportion of people (10.3% of men and 7.0% of women) perceive themselves to have difficulty controlling their sexual feelings, urges, and behaviors in a way that causes distress and/or impairment in their psychosocial functioning. A more plausible explanation is that the individuals who met the clinical cut point of the CSBI-13 capture the entire range of CSB, ranging from problematic but nonclinical out-of-control sexual behavior to the clinical diagnosis of CSBD. This suggests that the clinically relevant levels of distress and impairment associated with difficulty controlling one’s sexual feelings, urges, and behaviors may represent both a sociocultural problem and a clinical disorder (ie, a manifestation of sociocultural and intrapersonal conflicts around sexual values vs a clinical diagnosis of CSBD). Thus, health care professionals should be alert to the high number of people who are distressed about a lack of control over their sexual behavior and carefully assess the nature of the problem, consider its possible etiology, and find appropriate treatments for both men and women.

Our findings indicate that gender differences in endorsing clinically relevant levels of distress and impairment associated with difficulty controlling one’s sexual feelings, urges, and behaviors were much smaller than previously hypothesized.^20,21^ Men evidenced only a 54% greater likelihood (OR, 1.54; 95% CI, 1.15-2.06) of meeting the clinical cut point than women, who accounted for 41% of the sample who met the clinical screen cut point. Explanations justifying the hypothesis that CSBD may be much more common among men than women have been vague, although some researchers have pointed to differences in male sexuality with regard to intrinsic sexual motivation, ease of arousal, and more permissive attitudes toward casual sex.^4^ Such explanations tap into the sociosexual culture that underlies conceptualizations of masculine ideology (ie, male sexuality as “irrepressible”^22^) and suggest that when men get more access to sexual “outlets,”^22^ they may be more prone to developing compulsive sexual behavior. This is in contrast to feminine ideology that marks women as the “sexual gatekeepers,”^22^ who are expected to keep sexual urges in check and, thus, would be less likely to develop compulsive sexual behavior.

Given recent cultural shifts toward becoming more permissive of female sexual expression and the proliferation in accessibility to sexual imagery and casual sex through the internet, software applications, and social media, one possible explanation for the smaller gender differences found in our study is that the prevalence of difficulty controlling sexual behaviors among women may be increasing. Such an explanation warrants further empirical evaluation, given the lack of prior epidemiological estimates. Alternatively, given the dearth of data on CSBD among women, another possibility is that gender differences are truly much smaller than hypothesized. Researchers and clinicians are not immune to sociocultural biases regarding gender and sexual ideology^23^ and may therefore be more likely to overlook female CSBD or conceptualize it as a manifestation of another clinical issue (eg, trauma, bipolar, or borderline personality disorder).^24^ Future research should examine the myriad questions raised by this finding by examining longitudinal data, gender ideology and adherence to gender norms, and concomitant psychopathology.

With regard to demographic characteristics, we found that individuals with lower education, those with very high or very low income, racial/ethnic minorities, and sexual minorities were more likely to meet the clinical cut point than individuals who reported having higher education, having moderate income, and being white and heterosexual. These findings suggest the importance of understanding the sociocultural context in which distress surrounding difficulty controlling one’s sexual behavior occurs. However, we are aware of few studies to date that have examined the sociocultural context of CSBD, with the exception of sexual orientation.^13,25^ Researchers have argued that sexual minority men may be more at risk to develop sexual compulsivity, given their higher numbers of sexual partners, greater permissiveness of casual sex, and access to a variety of sexual outlets.^25^ More recently, however, research has found that minority stress increases risk for sexual compulsivity,^26^ and associated syndemic problems (eg, depression, anxiety, childhood sexual abuse, substance abuse, intimate partner violence, and sexual risk behavior) increase such risk among sexual minority men in a dose-dependent fashion.^27^ Our results corroborate the notion that minority stress increases risk for CSBD and suggests additional potential health disparities in CSBD. Hence, CSBD should not be assessed outside of its sociocultural context, and a public health approach may be warranted to address CSB.

### Limitations

The current study was limited by the nature of the survey and its methods. First, the CSBI-13 is a screening tool and has evidenced measurement error in its accuracy to distinguish the probable CSB clinical syndrome. Even if we account for scale measurement error (based on the 79% accuracy of the CSBI-13), the estimate (8.6%) remains higher than previously speculated and higher than that of other mental health problems (eg, prevalence of any depressive disorder is 5.7%^28^). Additionally, the NHSSB did not assess additional causes of distress about participants’ sexual behavior beyond lack of control, which limited our ability to interpret the meaning of the high prevalence rate. Erotic conflicts related to sociocultural norms about sexuality and gender, sexual orientation conflicts, and certain psychological disorders (eg, bipolar disorder, substance use problems, obsessive-compulsive disorder) that have been associated with sexual compulsivity may explain the presence of CSBD. This represents an important avenue for future research. Finally, this study could not rule out whether sociodemographic differences were due to scale bias. However, the possibility of scale bias is mitigated by the myriad versions of the CSBI that have been translated, validated, and studied in diverse populations within and outside of the United States.

## Conclusions

This study was the first we know of to document the US national prevalence of distress associated with difficulty controlling one’s sexual thoughts, feelings, and behaviors—the key feature of CSBD. The high prevalence of this sexual symptom has major public health relevance as a sociocultural problem and indicates a significant clinical problem that warrants attention from health care professionals. Moreover, gender, sexual orientation, race/ethnicity, and income differences suggest potential health disparities, point to the salience of sociocultural context of CSBD, and argue for a treatment approach that accounts for minority health, gender ideology, and sociocultural norms and values surrounding sexuality and gender. Health care professionals should be alert to the high number of people who are distressed about their sexual behavior, carefully assess the nature of the problem, and find appropriate treatments for both men and women.

## References

[1] Lee C The sex addiction epidemic. *Newsweek.* November 25, 2011 https://www.newsweek.com/sex-addiction-epidemic-66289. Accessed September 7, 2018.

[2] Braun-Harvey D, Vigorito MA Treating Out of Control Sexual Behavior: Rethinking Sex Addiction. New York, NY: Springer Publishing Co; 2015.

[3] Coleman E Is your patient suffering from compulsive sexual behavior? Psychiatr Ann. 1992;22(6):-. doi:10.3928/0048-5713-19920601-09

[4] Kafka MP Hypersexual disorder: a proposed diagnosis for *DSM-V*. Arch Sex Behav. 2010;39(2):377-400. doi:10.1007/s10508-009-9574-7 19937105

[5] Carnes P Out of the Shadows: Understanding Sexual Addiction. Center City, MN: Hazelden Publishing; 2001.

[6] Kaplan MS, Krueger RB Diagnosis, assessment, and treatment of hypersexuality. J Sex Res. 2010;47(2):181-198. doi:10.1080/00224491003592863 20358460

[7] Kraus SW, Krueger RB, Briken P, Compulsive sexual behaviour disorder in the *ICD-11*. World Psychiatry. 2018;17(1):109-110. doi:10.1002/wps.20499 29352554 PMC5775124

[8] Skegg K, Nada-Raja S, Dickson N, Paul C Perceived “out of control” sexual behavior in a cohort of young adults from the Dunedin Multidisciplinary Health and Development Study. Arch Sex Behav. 2010;39(4):968-978. doi:10.1007/s10508-009-9504-8 19421850

[9] Coleman E, Swinburne Romine R, Dickenson J, Miner MH Compulsive Sexual Behavior Inventory–13 In: Milhausen RR, Sakaluk JK, Fisher TD, Davis CM, Yarber WL, eds. Handbook of Sexuality-Related Measures. New York, NY: Routledge. In press.

[10] Coleman E, Miner M, Ohlerking F, Raymond N Compulsive sexual behavior inventory: a preliminary study of reliability and validity. J Sex Marital Ther. 2001;27(4):325-332. doi:10.1080/009262301317081070 11441516

[11] Miner MH, Raymond N, Coleman E, Swinburne Romine R Investigating clinically and scientifically useful cut points on the Compulsive Sexual Behavior Inventory. J Sex Med. 2017;14(5):715-720. doi:10.1016/j.jsxm.2017.03.255 28499521 PMC5472451

[12] Dodge B, Herbenick D, Fu T-C, Sexual behaviors of US men by self-identified sexual orientation: results from the 2012 National Survey of Sexual Health and Behavior. J Sex Med. 2016;13(4):637-649. doi:10.1016/j.jsxm.2016.01.015 26936073

[13] Coleman E, Horvath KJ, Miner M, Ross MW, Oakes M, Rosser BRS; Men’s INTernet Sex (MINTS-II) Team Compulsive sexual behavior and risk for unsafe sex among internet using men who have sex with men. Arch Sex Behav. 2010;39(5):1045-1053. doi:10.1007/s10508-009-9507-5 19588239 PMC3719393

[14] Miner MH, Coleman E, Center BA, Ross M, Rosser BRS The compulsive sexual behavior inventory: psychometric properties. Arch Sex Behav. 2007;36(4):579-587. doi:10.1007/s10508-006-9127-2 17192832

[15] McBride KR, Reece M, Sanders SA Predicting negative outcomes of sexuality using the Compulsive Sexual Behavior Inventory. Int J Sex Health. 2008;19(4):51-62. doi:10.1300/J514v19n04_06

[16] Storholm ED, Fisher DG, Napper LE, Reynolds GL, Halkitis PN A psychometric analysis of the Compulsive Sexual Behavior Inventory. Sex Addict Compulsivity. 2011;18(2):86-103. doi:10.1080/10720162.2011.584057 PMC10010680 36919045

[17] de Tubino Scanavino M, Ventuneac A, Rendina HJ, Sexual Compulsivity Scale, Compulsive Sexual Behavior Inventory, and Hypersexual Disorder Screening Inventory: translation, adaptation, and validation for use in Brazil. Arch Sex Behav. 2016;45(1):207-217. doi:10.1007/s10508-014-0356-5 25348356

[18] Træen B, Noor SW, Hald GM, Examining the relationship between use of sexually explicit media and sexual risk behavior in a sample of men who have sex with men in Norway. Scand J Psychol. 2015;56(3):290-296. doi:10.1111/sjop.12203 25688731 PMC5697722

[19] US Census Bureau and Bureau of Labor Statistics Current population survey. https://www.census.gov/programs-surveys/cps.html. Accessed January 18, 2018.

[20] Kafka MP What happened to hypersexual disorder? Arch Sex Behav. 2014;43(7):1259-1261. doi:10.1007/s10508-014-0326-y 24951045

[21] Kuzma JM, Black DW Epidemiology, prevalence, and natural history of compulsive sexual behavior. Psychiatr Clin North Am. 2008;31(4):603-611. doi:10.1016/j.psc.2008.06.005 18996301

[22] Tolman DL, Davis BR, Bowman CP “That’s just how it is”: a gendered analysis of masculinity and femininity ideologies in adolescent girls’ and boys’ heterosexual relationships. J Adolesc Res. 2016;31(1):3-31. doi:10.1177/0743558415587325

[23] Carvalho J, Guerra L, Neves S, Nobre PJ Psychopathological predictors characterizing sexual compulsivity in a nonclinical sample of women. J Sex Marital Ther. 2015;41(5):467-480. doi:10.1080/0092623X.2014.920755 24836276

[24] Ferree MC Females and sex addiction: myths and diagnostic implications. Sex Addict Compulsivity. 2001;8(3-4):287-300. doi:10.1080/107201601753459973

[25] Parsons JT, Kelly BC, Bimbi DS, DiMaria L, Wainberg ML, Morgenstern J Explanations for the origins of sexual compulsivity among gay and bisexual men. Arch Sex Behav. 2008;37(5):817-826. doi:10.1007/s10508-007-9218-8 17882541

[26] Rooney BM, Tulloch TG, Blashill AJ Psychosocial syndemic correlates of sexual compulsivity among men who have sex with men: a meta-analysis. Arch Sex Behav. 2018;47(1):75-93. doi:10.1007/s10508-017-1032-3 28840435

[27] Parsons JT, Rendina HJ, Moody RL, Ventuneac A, Grov C Syndemic production and sexual compulsivity/hypersexuality in highly sexually active gay and bisexual men: further evidence for a three group conceptualization. Arch Sex Behav. 2015;44(7):1903-1913. doi:10.1007/s10508-015-0574-5 26081246 PMC4561029

[28] World Health Organization *Depression and Other Common Mental Disorders: Global Health Estimates* Geneva, Switzerland: World Health Organization; 2017. http://www.who.int/mental_health/management/depression/prevalence_global_health_estimates/en/. Accessed September 7, 2018.

